# Embryonic thermal manipulation impacts the postnatal transcriptome response of heat-challenged Japanese quails

**DOI:** 10.1186/s12864-021-07832-7

**Published:** 2021-06-30

**Authors:** Anaïs Vitorino Carvalho, Christelle Hennequet-Antier, Aurélien Brionne, Sabine Crochet, Justine Jimenez, Nathalie Couroussé, Anne Collin, Vincent Coustham

**Affiliations:** 1 INRAE, Université de Tours, BOA, 37380 Nouzilly, France; 2 IFCE, INRAE, CNRS, Université de Tours, PRC, 37380 Nouzilly, France; 3 INRAE, LPGP, 35000 Rennes, France; 4 Université de Pau et des Pays de l’Adour, INRAE, NUMEA, E2S UPPA, 64310 Saint- Pée-sur-Nivelle, France

**Keywords:** Thermal manipulation, Temperature, Transcriptome, Avian, Embryonic treatment

## Abstract

**Background:**

The thermal-manipulation (TM) during egg incubation is a cyclic exposure to hot or cold temperatures during embryogenesis that is associated to long-lasting effects on growth performance, physiology, metabolism and temperature tolerance in birds. An increase of the incubation temperature of Japanese quail eggs affected the embryonic and post-hatch survival, growth, surface temperatures and blood characteristics potentially related to thermoregulation capacities. To gain new insights in the molecular basis of TM in quails, we investigated by RNA-seq the hypothalamus transcriptome of 35 days-old male and female quails that were treated by TM or not (C, control) during embryogenesis and that were exposed (HC) or not (RT) to a 36 °C heat challenge for 7 h before sampling.

**Results:**

For males, 76, 27, 47 and 0 genes were differentially expressed in the CHC vs. CRT, CRT vs. TMRT, TMHC vs. TMRT and CHC vs. TMHC comparisons, respectively. For females, 17, 0, 342 and 1 genes were differentially expressed within the same respective comparisons. Inter-individual variability of gene expression response was observed particularly when comparing RT and HC female animals. The differential expression of several genes was corroborated by RT-qPCR analysis. Gene Ontology functional analysis of the differentially expressed genes showed a prevalent enrichment of terms related to cellular responses to stimuli and gene expression regulation in both sexes. Gene Ontology terms related to the membrane transport, the endoplasmic reticulum and mitochondrial functions as well as DNA metabolism and repair were also identified in specific comparisons and sexes.

**Conclusions:**

TM had little to no effect on the regulation of gene expression in the hypothalamus of 35 days-old Japanese quails. However, the consequences of TM on gene expression were revealed by the HC, with sex-specific and common functions altered. The effects of the HC on gene expression were most prominent in TM females with a ~ 20-fold increase of the number of differentially expressed genes, suggesting that TM may enhance the gene response during challenging conditions in female quail hypothalamus. TM may also promote new cellular strategies in females to help coping to the adverse conditions as illustrated by the identification of differentially expressed genes related to the mitochondrial and heat-response functions.

**Supplementary Information:**

The online version contains supplementary material available at 10.1186/s12864-021-07832-7.

## Background

The avian thermal-manipulation (TM) procedure during embryogenesis is an embryonic treatment (ET) consisting in modifying the egg incubation temperature, generally in a cyclic manner and during a specific window of embryonic development [1]. TM has been extensively studied in chickens [2–10] and in other bird species such as turkeys [11, 12], ducks [13–16] and quails [17–19]. TM in chickens was generally associated with long-lasting effects on the growth performance (weight gain, muscle yield, etc.), the physiology (thyroid axis function, acid-base balance, respiratory process, etc.) and the metabolism (glucose metabolism, regulation of mitochondrial function, etc.) [1]. Moreover, TM corresponding to a cyclic increase of egg incubation temperature was shown to improve the heat tolerance of male broilers exposed to a heat challenge at slaughter age [10], opening promising avenues to allow chickens better coping with high temperatures in a context of global warming. Beyond its agronomic interests, TM is also a valuable scientific tool to explore the molecular mechanisms involved in the long-term memory of an embryonic environmental exposure in vertebrates. TM was recently shown to modify muscle transcriptome [3] and hypothalamus epigenome [20] of broiler chickens, suggesting that an epigenetic regulation of gene expression may be involved in the phenotypic responses of TM. However, knowledge on the mechanisms underlying TM remains scarce, and have yet to be explored in other avian species to uncover core molecular mechanisms that may be relevant for many bird species and beyond.

In addition to being an agronomic species of interest for its meat and eggs, the Japanese quail (*Coturnix japonica*) is an avian model closely related to chickens that is popular in developmental biology [21] as well as in genetic and genomic analyses [22–24]. We showed in a previous study that TM has short and long-term effects on the development and the physiology of the Japanese quail [25]. TM in quails consisted in an increase of 1.7 °C of the incubation temperature, applied from the 12th hour until the 13th day of incubation, 12 h per day. Heat tolerance was tested by a heat exposure of 36 °C (vs. 22 °C) for 7 h at 35 days of age (D35) but this moderate challenge did not reveal a clear improvement of heat resistance in TM quails [25]. However, among other changes, surface temperatures of legs and beak were affected by TM, the latter in interaction with sex and the heat challenge at D35, suggesting that TM may affect the way the quails are able to dissipate the heat [25].

To gain new insights into the molecular mechanisms involved in the long-lasting impacts of TM in quails, we investigated the impact of TM on the transcriptome of quails that were exposed or not to a heat challenge at D35 as previously described [25]. At the difference of the previous study in chickens for which only males were considered [3], we explored the transcriptome of both female and male quails, as our phenotypic data showed sex-specific phenotypic responses [25]. This current study focused on the hypothalamic transcriptome given its central role in the thermoregulation, the demonstration of an epigenetic impact of TM in chicken hypothalamus [20] and its importance in the molecular mechanisms of the post-natal thermotolerance acquisition in chicks [1, 26], another perinatal heat conditioning strategy in birds.

## Results

### Identification of hypothalamic differentially expressed genes by RNA-seq resulting from the embryonic and D35 treatments

**Fig. 1:**
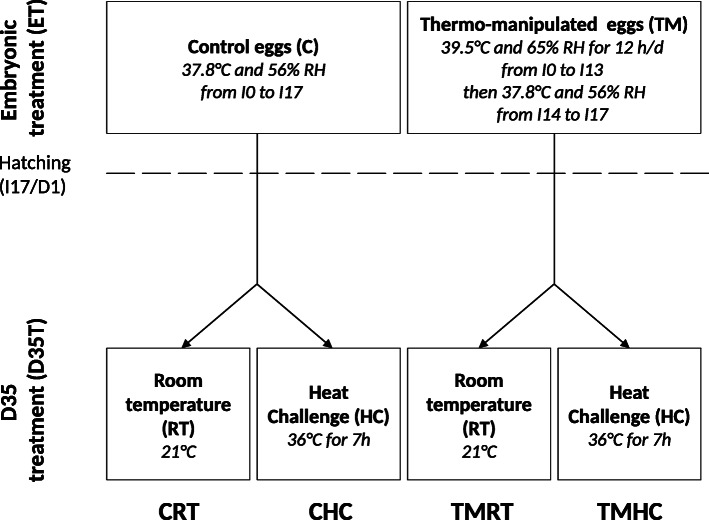
Summary of the experimental procedure. Eggs were randomly incubated either in control (C) or in thermal-manipulation (TM) conditions from incubation days I0 to I13. After hatching, all quails were reared collectively until the age of 35 days (D35). At D35, animals from each embryonic group were separated in two D35 treatment groups: either they were left at room temperature (RT) conditions for 7 h, or they were heat challenged (an increase of the environmental temperature at 36 °C) for 7 h

**Fig. 2:**
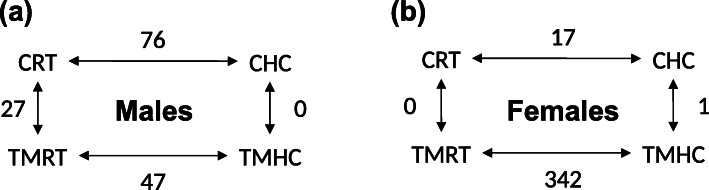
Number of differential expressed genes (DEG) obtained in each pairwise comparison. (**a**) DEG numbers for males. (**b**) DEG number for females. CRT: Control incubation followed by a room temperature treatment at D35; CHC: Control incubation followed by a heat challenge treatment at D35; TMRT: Thermal manipulation during incubation followed by a room temperature treatment at D35; TMHC: Thermal manipulation during incubation followed by a heat challenge treatment at D35

**Fig. 3:**
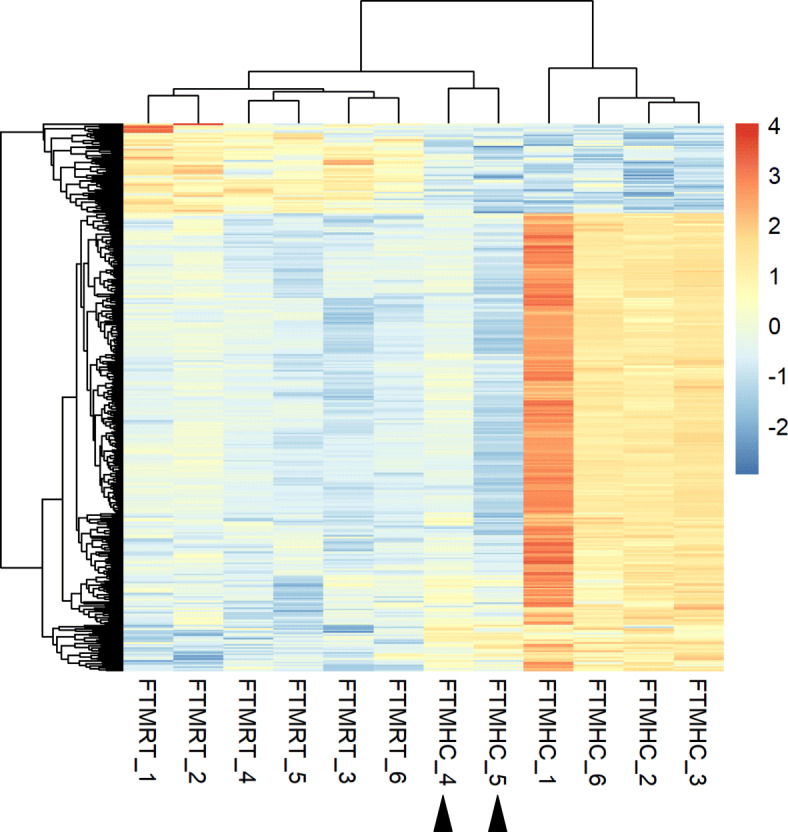
Heat map of the RNA-seq analysis based on the 342 DEG from the female TMHC vs. TMRT comparison. The six biological replicates per treatment are shown below the heat map. The arrows point to the two TMHC females that cluster with the six TMRT females. TMHC: Thermal manipulation during incubation followed by a heat challenge treatment at D35; TMRT: Thermal manipulation during incubation followed by a room temperature treatment at D35

The RNA-sequencing of the 48 hypothalamic samples obtained from the four experimental conditions (i.e. CRT, CHC, TMRT and TMHC; Fig. 1) in both sexes generated in average 30.7 +/- 7 million reads per sample (Additional file 1). An average of 90.4 % +/- 2.4 of the reads were uniquely aligned to the reference quail genome, version 2.0 (Additional file 2). Reads were counted to genes using the reference quail annotation and differentially expressed genes (DEG) were identified by pairwise comparisons of the four experimental conditions from female and male datasets separately (Fig. 2, Additional file 3 and deposited data). In males, 76, 27 and 47 genes were determined as DEG in CHC vs. CRT, CRT vs. TMRT and TMHC vs. TMRT comparisons respectively. No DEG was identified between CHC and TMHC male quails (Fig. 2a, Additional file 4). In females, no DEG was identified between CRT and TMRT animals. However, 17, 342 and 1 genes were found differential in the CHC vs. CRT, TMHC vs. TMRT and CHC vs. TMHC comparisons (Fig. 2b and Additional file 4). To get a sense of the inter-individual variability given the relatively low number of DEG identified, a heat map was generated from the 342 DEG from the TMHC vs. TMRT comparison observed in females using log2 counts-per-million (Fig. 3). Interestingly, two female TMHC individuals (pointed by an arrow in Fig. 3) were clustered with the other TMRT females. These two TMHC females also appeared separated from other TMHC females in the multidimensional scaling analysis of the RNA-seq data (Additional file 5, panel a, pointed by an ellipse).

### Analysis of common DEG between treatments and sexes

**Fig. 4:**
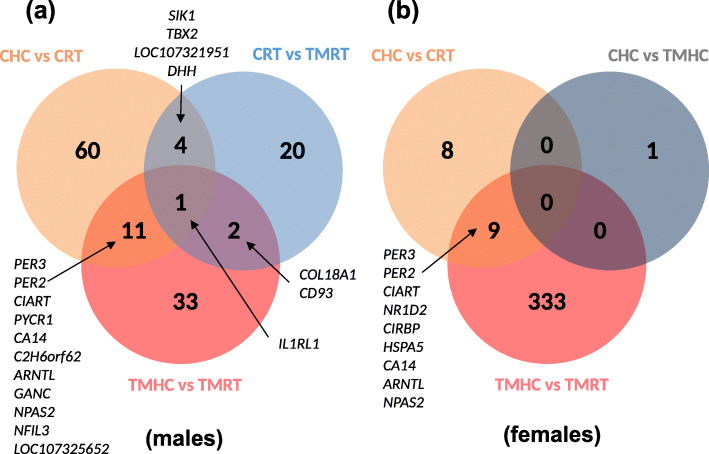
Venn diagram showing common and specific genes between differential expressed genes (DEG) lists. (**a**) Venn diagram for males, and (**b**) for females. CRT: Control incubation followed by a room temperature treatment at D35; CHC: Control incubation followed by a heat challenge treatment at D35; TMRT: Thermal manipulation during incubation followed by a room temperature treatment at D35; TMHC: Thermal manipulation during incubation followed by a heat challenge treatment at D35

The comparison of the DEG lists between experimental conditions from female and male datasets revealed little overlap (Fig. 4). In the male datasets, the *IL1R1* gene coding for interleukin 1 receptor was the only one found in common between the three DEG lists (CHC vs. CRT, CRT vs. TMRT and TMHC vs. TMRT; Fig. 4a). Eleven genes (*PER3, PER2, CIART, PYCR1, CA14, C2H6orf62, ARNTL, GANC, NPAS2, NFIL3* and *LOC107325652*) were impacted by the HC irrespectively of the embryonic treatment as they were found in both CHC vs. CRT and TMHC vs. TMRT comparisons. Additionally, four genes (*SIK1, TBX2, LOC107321951* and *DHH*) were found in both CHC vs. CRT and CRT vs. TMRT comparisons. Two genes (*COL18A1* and *CD93*) were found in both CRT vs. TMRT and TMHC vs. TMRT comparisons. In the female datasets, given the low number of DEG identified except for the TMHC vs. TMRT comparison, only the comparison CHC vs. CRT and TMHC vs. TMRT had nine common DEG (*PER3, PER2, CIART, NR1D2, CIRBP, HSPA5, CA14, ARNTL* and *NPAS2*; Fig. 4b).

Interestingly, some DEG were shared by both female and male datasets (Additional file 6). For both CHC vs. CRT and TMHC vs. TMRT comparisons, *PER3, PER2, CIART, CA14, ARNTL* and *NPAS2* were found differential for both sexes. Three other DEG were found in both sexes for each comparison: *ACAN*, *NR1D2* and *NFIL3* for the CHC vs. CRT comparison; *SLC13A5*, *CIRBP* and *LOC107313685* (coding for a putative melatonin receptor type 1 A according to NCBI database) for the TMHC vs. TMRT comparison. Finally, eight other genes (*CD320*, *LOC107310113*, *TEF*, *PMM1*, *MED11*, *LOC107312385*, *LOC107307123* and *OGG1*) were DEG in both sexes irrespectively of the comparison (Additional file 3).

### RT-qPCR analysis of selected DEG

**Fig. 5:**
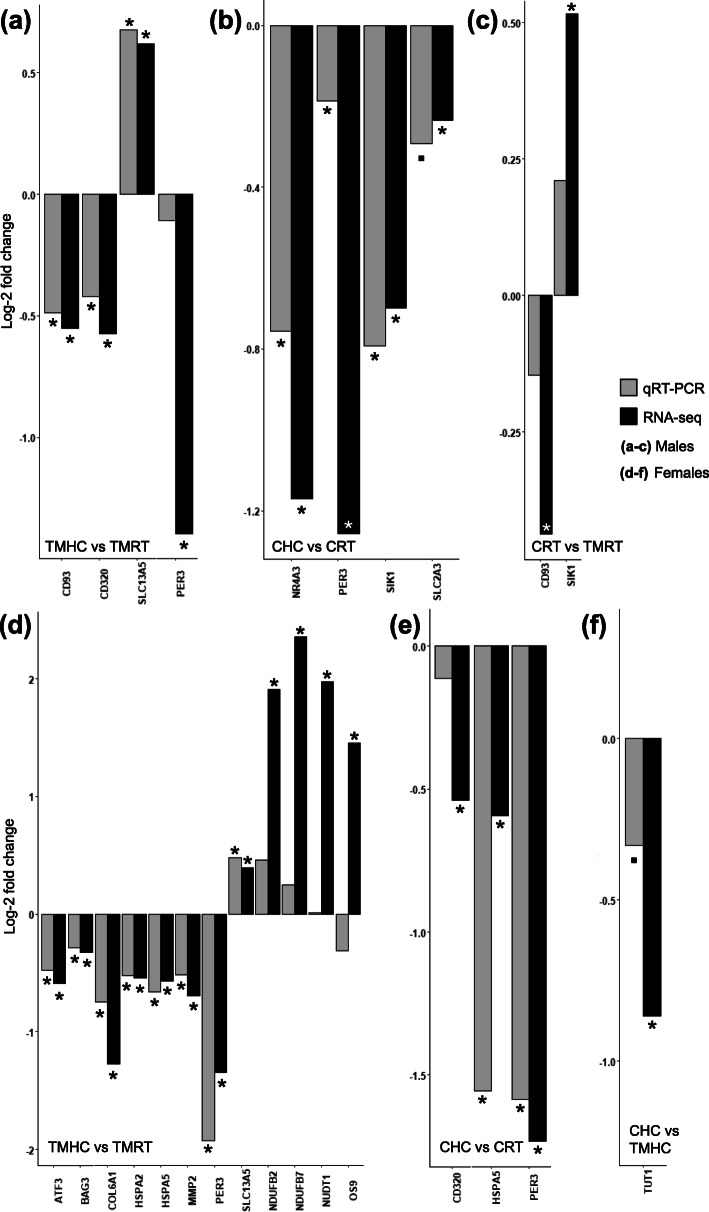
Graphic showing the gene expression results obtained by RT-qPCR and RNA-sEq. (**a**-**c**) Log 2-transformed fold changes of genes identified in the three following male comparisons: (**a**) TMHC vs. TMRT, (**b**) CHC vs. CRT and (**c**) CRT vs. TMRT. (**d**-**f**) Log 2-transformed fold changes of genes identified in the three following female comparisons: (**d**) TMHC vs. TMRT, (**e**) CHC vs. CRT and (**f**) CHC vs. TMHC. Grey bars represent RT-qPCR data, black bars represent RNA-seq data. When the difference is significant, p-values are shown as follows: ⁕ *p* ≤ 0.05; • *p* ≤ 0.1

RT-qPCR was used to measure the level of expression of genes identified as DEG in a larger set of individuals (including individuals used for the RNA-seq experiment; Fig. 5). All RT-qPCR analyses were performed on the comparisons that led to the identification of DEG in the transcriptome analysis.

Seven candidates were randomly selected from the DEG analysis for the males (Fig. 5a-c). For the TMHC vs. TMRT comparison, four genes were tested by RT-qPCR: *CD93, CD320, SLC13A5* and *PER3*. All but *PER3* were significantly DEG in the RT-qPCR assay and all genes had similar fold changes (Fig. 5a). The same result was found for the CHC vs. CRT comparison, as three candidate genes (*NR4A3, PER3, SIK1*) out of four (i.e. with the exclusion of *SLC2A3*) were differentially expressed in the RT-qPCR assay with equivalent fold changes compared to the RNA-seq analysis (Fig. 5b). However, regarding the CRT vs. TMRT comparison, RT-qPCR did not corroborate the transcriptome findings as both *CD93* and *SIK1* genes were not DEG despite consistent directions of changes of expression with the RNA-seq analysis (Fig. 5c).

Fourteen candidates were randomly selected from the DEG analysis for the females (Fig. 5d-f). A set of 12 genes from the TMHC vs. TMRT comparison was tested by RT-qPCR (Fig. 5d). All but *OS9* gene showed the same direction of variation in both analyses. Eight genes were found differential between TMCH and TMRT conditions in the RT-qPCR assay (*ATF3, BAG3, COL6A1, HSPA2, HSPA5, MMP2, PER3* and *SLC13A5*). In contrast, *NDUFB2, NDUFB7*, *NUDT1* and *OS9* were not found differential in the RT-qPCR analysis. Regarding the 3 genes selected from the CHC vs. CRT comparison (*CD320*, *HSPA5* and *PER3*; Fig. 5e), the direction of the variation found by RT-qPCR and RNA-seq was consistent and the difference of expression was significant for all but *CD320*. Finally, the expression level of *TUT1* gene from the CHC vs. TMHC comparison was also tested in females (Fig. 5f) but no differential expression was found by RT-qPCR for this gene despite similar direction of variation.

### Gene Ontology functional analysis of DEG lists

**Fig. 6:**
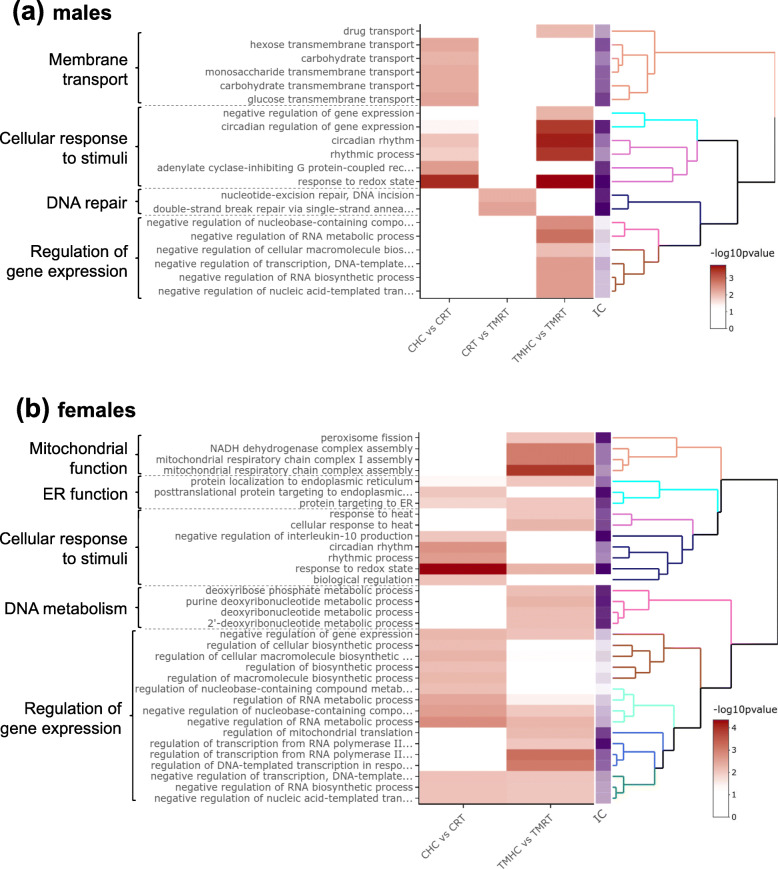
Gene Ontology functional analysis of the differentially expressed genes lists. The clustering heat map plots of the functional sets of gene ontology (GO) terms were obtained using ViSEAGO. (**a**) Gene Ontology functional analysis in males. (**b**) Gene Ontology functional analysis in females. From left to right are shown the major processes, the enriched GO terms, a heat map (in red tones) showing –log10 p-value of enrichment test, a heat map (in purple tone) showing information content and a dendrogram on enriched GO terms based on BMA semantic similarity distance and Ward’s clustering criterion. CRT: Control incubation followed by a room temperature treatment at D35; CHC: Control incubation followed by a heat challenge treatment at D35; TMRT: Thermal manipulation during incubation followed by a room temperature treatment at D35; TMHC: Thermal manipulation during incubation followed by a heat challenge treatment at D35. IC: Information Content

In order to reveal biological processes impacted by both embryonic and D35 treatments, lists of DEG from male and female datasets (except for the comparison CHC vs. TMHC in females with only 1 DEG) were analysed with the ViSEAGO R package [27] by hierarchical clustering enriched Gene Ontology (GO) terms (Fig. 6 and Additional file 7). In this analysis, four and five broad functions were identified in males and females, respectively.

In males, 20 GO terms were found significantly enriched in the analysis. The analysis in non-challenging conditions (CRT vs. TMRT) led to the identification of two specifically enriched GO terms related to the DNA repair-related processes in males. GO terms related to the membrane transport and the cellular responses to stimuli (in particular, rhythmic processes) were significantly enriched in both CHC vs. CRT and TMHC vs. TMRT groups (Fig. 6a). The effect on membrane transport seemed however prevalent in controls (5 GO terms) compared to TM (1 GO term). Moreover, six GO terms related to the regulation of gene expression and RNA metabolism were significantly enriched in response to the HC in TM animals only.

In females, 34 GO terms were found significantly enriched in the two comparisons included in the analysis that reflect the HC response in both TM and control animals (CHC vs. CRT and TMHC vs. TMRT; Fig. 6b). Enriched GO terms related to the mitochondrial function (4 terms), the endoplasmic reticulum function (3 terms), the cellular response to stimuli (7 terms) and the regulation of gene expression through RNA metabolism (16 terms) were found in both comparisons. GO terms related to DNA metabolism (4 terms) were specific to the TMHC vs. TMRT comparison in females.

When comparing ViSEAGO results from both sexes, the analysis revealed that clusters related to the cellular response to stimuli and gene expression regulation seemed to be affected by the HC in both sexes, despite the latter being only found in the TM comparison in males. This finding is supported by 9 enriched GO terms found in both sexes related either to the cell response (GO:0007623, GO:0048511 and GO:0051775) or to the RNA metabolism (GO:0010629, GO:0045934, GO:0051253, GO:0045892, GO:1,902,679 and GO:1,903,507).

## Discussion

To gain new insights into the molecular basis of the effects of TM in the Japanese quail, we performed a transcriptome analysis of hypothalamic samples of 35 days-old male and female TM and C quails, exposed (HC) or not (RT) to a heat challenge [25]. In the present study, a RNA-seq investigation of the transcriptome was performed on six animals per experimental condition and sex to identify the main changes of gene expression as recommended in a recent extensive report [28]. Despite the fact that the relevance of gene validation of RNA-seq data by candidate approaches such as RT-qPCR remains debated [29], RT-qPCR assays revealed consistent changes in gene expression (Fig. 5), suggesting that the RNA-seq results are reproducible by another approach within the same experiment and therefore of good quality.

Analysis of the RNA-seq data showed that TM had little impact on the hypothalamus transcriptome with only 27 DEG identified in males and none in females (CRT vs. TMRT comparisons). Functional analysis of the 27 male DEG revealed only two significantly enriched GO terms related to the DNA repair function. This is consistent with the fact that heat exposure may be associated to DNA damage [30]. However, a particular care should be taken with this result as these two GO terms are defined by only one DEG, *OGG1* (Additional file 7, lines 14–15). In yeast, *OGG1* was shown to be involved in the repair of a pyrimidine and purine base lesions resulting from oxygen radicals during heat exposure [31]. Interestingly, *OGG1* was also identified as DEG in the female TMHC vs. TMRT comparison. Therefore, TM during embryogenesis may trigger DNA repair mechanisms through *OGG1* in both sexes.

The limited impact of TM in quail hypothalamus is similar to the impact of TM observed in a previous transcriptome investigation in male chickens that also showed limited changes in gene expression in the pectoral muscle [3]. Therefore, TM may have a relatively low impact on the gene expression in basal conditions in avian species, which is consistent with the few physiological traits impacted by TM at D35 in thermoneutral conditions [25]. Moreover, TM effects in quail transcriptome may be more important in the first weeks of life as suggested by the growth differences observed from hatching to 4 weeks of life and not beyond [25]. Thus, differences in gene expression in earlier developmental stages may have been missed and would be worth considering in future studies.

Previous work based on physiological analyses in quails revealed that thermal manipulation effects may be revealed by the presence of a heat challenge [25]. However, in standard incubation conditions (C), HC had a relatively low impact on the hypothalamic transcriptome in our study (76 DEG in males, 17 in females), which is surprising given that previous studies revealed thousands of probe sets impacted by heat stress in the hypothalamus transcriptome of chickens [32]. This discrepancy may be explained by the moderate effect that the HC had on quails in our study. Indeed, the 36 °C temperature increase for 7 h failed to trigger behavioural signs of distress related to heat such as panting, prostration, reduction of food intake or increase in water intake [25]. Moreover, chickens are likely to be less resistant to heat due to a more extensive genetic selection, especially for meat-type broiler chickens, which may lead to a stronger gene response [33]. Furthermore, little impact of HC was also observed in TM animals, with 47 DEG in males and 342 DEG in females. This may be explained, at least in part, by the inter-individual variability of the response to the heat challenge that we observed. Inter-individual variability is known to hinder the proper identification of environmental impacts in animals [34]. In our experiment, the heat map of the 342 DEG from the female TMHC vs. TMRT comparison showed a weaker response of two of the six TMHC females to the HC (Fig. 3). The MDS analysis also supported this observation with the two TMHC females colocalizing with the TMRT females. We verified that the two “non-responder” TM females and the four other TMHC females were phenotypically similar by visual inspection and weight measurement before sampling (222 and 302 g for the non-responders vs. 217, 212, 312 and 202 g for the 4 other TMHC females). Despite the fact that these two animals may arguably be considered as outliers, we chose not to remove them from the differential analysis as they represent the true variability that exists in response to a heat exposure. High variability was also observed in the RT-qPCR assays that failed to corroborate the RNA-seq data for five genes (*PER3*, *NDUFB2*, *NDUFB7*, *NUDT1* and *OS9*) among the 16 assayed (TMHC vs. TMRT comparisons in both sexes). In consequence, our analysis may have led to the identification of the most robust DEG while overlooking some of the most discreetly affected genes.

In C incubation conditions, the functional analysis showed that the HC affected genes related to the cellular response to stimuli in both sexes, to the membrane transport in males and to the ER function and the regulation of gene expression in females. The cluster related to the cellular response to stimuli contained mostly GO terms related to the clock and the regulation of the redox status in both sexes. The redox balance is a known component of the cellular response to heat [35], but genes defining these GO terms are also known regulators of the clock (*NPAS2* and *ARNTL*). However, the identification of GO terms related to the clock may be explained by an experimental bias due to the fact that RT animals were sampled in the morning (while HC animals were challenged) and that HC were sampled in the afternoon [25]. Thus, the question remains as to whether these DEG actually result from the HC and is not a consequence of the sampling time. Nonetheless, components of the circadian clock were recently shown to be involved in the heat stress response of the transcriptome in plants during the day [36]. Thus, the circadian response may still be a possible mechanism despite our experimental design does not allow us to conclude on that effect. Other functions appeared to be impacted in only one sex. In males, GO terms related to the membrane transport were significantly enriched, suggesting that cellular communication is altered by the heat challenge. This observation is consistent with the fact that heat stress is sensed by the rearrangement of plasma membrane components, which is signalled toward HSF1 activation and the expression of heat shock proteins [37] that are also found as DEG in our analysis (such as *HSPA2*, the orthologue of *Hsp70*). Furthermore, sugar transport seems particularly affected, as previously reported in the chicken intestine where a relationship between heat stress and apical glucose transport was observed [38]. In control females, HC altered genes related to the regulation of gene expression and RNA metabolism. RNA metabolism is a known player of temperature adaptation in plants [39] and may contribute through diverse biochemical properties to the thermosensing capacities and acclimatization in animals [40].

Interestingly, the heat challenge led to 47 and 342 DEG in males and female TM quails, respectively, in contrast to the 76 and 17 DEG identified in C quails from the same respective sexes. Thus, as previously observed for the physiological traits analysed [25] and at the difference of chickens for which studies mostly focused on males [1], female quails seemed more affected by the TM than the males when exposed to heat in later life. This result suggests that TM has a long lasting impact on gene expression, especially in females, which is revealed under elevated temperature conditions. This result is similar to the previous observation of a ~ 5 times enhanced gene response in the muscle transcriptome of male TM chickens that were exposed to the HC at D35 compared to animals from a standard incubation challenged at the same age [3]. Altogether, both studies are consistent with the hypothesis of a TM-induced hormetic temperature-priming [25] that may be widespread in birds in both peripheral and central tissues.

Functional analyses in TMHC vs. TMRT quails revealed similarities but also differences compared to what was found in the CHC vs. CRT analysis. In males, both functions related to membrane transport and cellular response to stimuli are found in the TMHC vs. TMRT comparisons despite only one GO term is related to the membrane transport. The finding of a redox/clock effect, like in control quails, is consistent with the fact that the same sampling bias exists in the TM group (see above). Interestingly, genes functioning in the regulation of gene expression and RNA metabolic processes were only identified in the TMHC vs. TMRT comparison in males, and not in the C comparison, suggesting that TM triggers gene regulation programs in males exposed to heat several weeks post-hatching. In females, functions related to the cellular response to stimuli and the regulation of gene expression were identified in the TMHC vs. TMRT comparison, similarly to the C females and, to some extent, to the males (regarding the clock and gene expression in the TM group). However, concerning the cellular response to stimuli function, beyond the GO term related to the response to redox state, two GO terms specific to the female TM comparison were related to the response to heat (defined by 3 genes: *CPLB, BAG3* and *IER5*). The co-chaperone gene *BAG3*, that was validated by RT-qPCR in our assay (Fig. 5), regulates cellular adaptive responses against stressful conditions and was previously shown to affect the nucleocytoplasmic shuttling of the heat shock factor HSF1, the master transcriptional regulator of chaperone genes, upon heat stress [25]. Heat exposure was also shown to induce a robust expression of IER5, a regulator of the cell proliferation, in a heat shock factor HSF1-dependent manner [25]. Thus, HC in TM females is likely to trigger an adaptive gene response related to HSF1 that may play a protective role by ensuring proper folding and distribution of proteins within hypothalamic cells. Moreover, specifically for the TMHC vs. TMRT female comparison, functions related to the mitochondria (the powerhouse of the cell) and the DNA metabolism were altered. In male broilers, TM was shown to impact the expression of several mitochondrial genes in thermo-neutral conditions such as *MRPL28* and *COQ6*, and many more genes related to the mitochondrial function when comparing TMHC and TMRT animals [3]. In our study, genes related to the mitochondrial function appeared to be solely affected by the heat exposure in females, which may be explained by a species-specific response, a difference in the treatment, the sensitivity of the gene response or the different tissue analysed. Moreover, GO terms related to the metabolism of DNA observed in females TMHC vs. TMRT were defined by 2 genes: *NUDT1* (however not validated by RT-qPCR) and *NUDT18*. This last gene encodes a hydrolase that helps maintain the high fidelity of DNA replication and transcription under oxidative stress. As oxidative stress may be triggered in birds by acute heat exposure [43, 44], this response could contribute to alleviating some of deleterious effects of heat-induced oxidative injuries on the hypothalamic transcription process.

Altogether, the functional analysis revealed a similar impact of the HC in C and TM animals with additional functions affected in TM quails in response to the HC related to the regulation of gene expression in males and to the heat production and response in females. Given the GO terms related to the regulation of gene expression found in all HC vs. RT comparisons except in C males, it is possible that both sex (female) and ET (TM) factors contribute to enhance the response of genes related to the regulation of gene expression induced by the HC. The enhanced gene response is also clearly illustrated in females by the ~ 20-fold increase of DEG in the TM comparison compared to the C comparison despite inter-individual variability seen in the response to heat. The mechanisms underlying the TM-induced enhanced gene response are unknown but a growing line of evidence suggest that they may be of epigenetic origin. Indeed, we previously showed that TM affected two histone marks, H3K4me3 and H3K27me3, in male chicken hypothalamus that are likely to be involved in the epigenetic memory of TM [20]. Therefore, TM may trigger “silent” epigenetic reprogramming of genes that permits gene expression changes under stressful conditions. Moreover, epigenetic mechanisms were shown to be involved in the post-natal thermal programming in birds that consists in modulating the ambient temperature of chicks during the first days of life to improve thermal tolerance of the animals. Notably, hypothalamic changes in DNA methylation and hydroxymethylation, histone marks and micro-RNA were shown to be involved in the postnatal acquisition of temperature resilience of chicks [41–43]. An investigation of the epigenetic status of the DEG would therefore be worth performing to gain a better understanding of the molecular mechanisms underlying TM-induced gene expression changes.

## Conclusions

By itself, TM had little to no impact on the regulation gene expression in the hypothalamus of the Japanese quail. However, the effects of TM on gene expression were revealed by the HC at D35, leading to both common and specific gene expression changes in males and females. The effects of TM on gene expression changes induced by HC were most prominent in females with a ~ 20-fold increase of the number of DEG. Thus, TM may enhance the gene response to stressful conditions in quail hypothalamus while promoting new cellular strategies to help coping to the adverse conditions as illustrated by the identification of DEG related to the mitochondrial and heat-response functions. Further work is now required to gain a better understanding of the molecular mechanisms, likely of epigenetic nature, potentially driving this variable and sex-specific enhanced response of TM quails exposed to heat in later life.

## Methods

### Experimental design

Animal rearing was performed in the PEAT INRAE Poultry Experimental Facility (2018, 10.15454/1.5572326250887292E12). The hypothalamic tissues used in this study were obtained from animals described in a previous study [25]. To summarize, eggs of Cons DD Japanese quail line [44] were collected during 12 days at 16 °C and dispatched between two embryonic treatments (ET): Control incubation group (C) or Thermo-Manipulated incubation group (TM). Eggs were incubated in two automated commercial incubators (one per treatment) with automated regulation of temperature, humidity and ventilation (Bekoto B64-S, Pont-Saint-Martin, France). C eggs were maintained at 37.8 °C and 56 % relative humidity (RH) during the whole incubation period from the start (I0) to day 13 of incubation (I13). TM eggs were incubated at 39.5 °C and 65 % RH for 12 h/d from I0 to I13 and then in the same condition than C (Fig. 1). At I14, all eggs were transferred to one unique hatcher and incubated at 37.5 °C with RH between 75 and 80 % until I17. The quails hatched without body defects at I17/D1 from the two experimental conditions (262 C and 243 TM) were transferred in a single concrete-floored room covered with litter and reared in standard conditions. At D25, all animals were sexed by visual inspection of the feathers. At D35, quails were dispatched between two D35 treatments: half (same proportion of C and TM animals) was kept under room temperature (RT − 81 CRT and 79 TMRT) and the other half was submitted to a heat challenge (HC - environmental temperature increase at 36 °C for 7 h – 71 CHC and 57 TMHC) (Fig. 1). The same proportion of animals from both sexes was considered in each experimental group. Samples from the RT quails were collected in the morning while HC animals were heat challenged. Thus, HC quails were sampled in the afternoon of the same day. Animals were slaughtered and the hypothalamus sampled on 9–10 representative animals randomly selected for each condition (CRT, CHC, TMRT or TMHC, with the same proportion of females and males).

### RNA extraction

RNA extraction was performed using the All Prep RNA/DNA Mini kit (Qiagen), according to the manufacturer’s instruction. RNA concentration was obtained on a Nanodrop ND-1000 UV-Vis Spectrophotometer. RNA integrity was verified by on an Agilent 2100 bioanalyzer with a RNA Nano kit (Agilent), according to the manufacturer’s instruction. All sample RIN were above 8.5.

### Library construction, RNA sequencing and bioinformatics analysis

Six biological replicates per experimental condition and sex were included in the RNA-seq experiment. Library construction and sequencing were performed the GenomEast genomic platform (IGBMC, Illkirch, France). Libraries were created from 500 ng of total RNA using the TruSeq stranded mRNA library prep kit and TruSeq RNA single indexes kits A and B (Illumina). Libraries were sequenced with an Illumina Hi-Seq 4000 sequencer using a multiplexing strategy (50 bp single-end reads). Image analysis and base calling were performed using RTA 2.7.3 and bcl2fastq 2.17.1.14. Sequenced reads were mapped to *Coturnix japonica 2.0* genome obtained from the NCBI database (Coturnix japonica 2.0, GCA_001577835.1) using STAR version 2.7.0a with default arguments [45]. Reads were counted using FeatureCounts version 1.6.3 with arguments -T 6 -s 2 -t exon -g gene [46]. The heat map was drawn using the pheatmap R function version 1.0.12 (https://CRAN.R-project.org/package=pheatmap), using log2 counts-per-million with normalized library sizes, Euclidean distance between genes and complete clustering method. The datasets supporting the conclusions of this article are available at the GEO repository GSE161976.

### Gene expression analysis by RT-qPCR

RT-qPCR was performed as previously described [47]. Nine to 10 animals per conditions and sex (including the one used for the RNA-seq experiment) were used. The cDNAs were synthesized from 500 ng of total RNAs using the Superscript II enzyme (Invitrogen) and hexamer random primers (Promega), following the manufacturer’s instructions. Quantitative real-time PCR (qPCR) was carried out with Takyon qPCR Kits (Eurogentec) and using a LightCycler® 480 Instrument II system (Roche) with 384-well plates (4TI-0382, 4titude).

Primer sequences were designed with NCBI Primer blast (https://www.ncbi.nlm.nih.gov/tools/primer-blast/index.cgi?LINK_LOC=BlastHome) software. Primer sequences are available in the Additional file 8. Three technical replicates were done for each sample and a standard curve protocol was used to evaluate gene expression. To assess the specificity of the amplification, every PCR product size was checked on a 2 % agarose gel and sequenced by Sanger sequencing (Genewiz). Relative expression was normalized to the expression of five or six reference genes (selected among 9 reference genes) according to the sex (*GAPDH*, *PGK1*, *RPS7*, *RPS8* and *YWHAZ* for males and *ACTB*, *GAPDH*, *PGK1*, *SDHA*, *TBP* and *YWHAZ* for females; Additional file 8) using the qbase + software (version 3.2, Biogazelle, Gent, Belgium).

### Statistical analyses

All statistical analyses were performed with R software, version 3.6.1 [48].

Statistical analyses of the RNA-seq data were performed using the Bioconductor edgeR package (version 3.24.3) [49, 50] to identify the differentially expressed genes (DEG) between the four experimental conditions CRT, CHC, TMRT and TMHC. Samples from females and males were analysed independently in two datasets. TMM normalization (trimmed mean of M values) was applied to each dataset to account for compositional difference between the libraries. A generalized linear model was fitted to test the pairwise comparisons between all four conditions with likelihood ratio tests. P-values were adjusted by controlling the false positive rate below 0.05 with a Benjamini-Hochberg correction [51]. Lists of DEG between all four experimental conditions from females and males datasets were compared using Venn diagram in jvenn web application [52].

Differentially expressed genes identified by RNA-seq were also investigated in RT-qPCR. Gene expression data obtained by RT-qPCR was transformed with a logarithmic function (base = 2) and differential expression between two biological conditions was analysed with Student’s test or Welch’s test upon assumption of equal variances. The difference in gene expression by RT-qPCR between conditions was considered significant for p-values ≤ 0.05.

### Gene Ontology functional analysis

Gene annotation was performed with the EntrezGene NCBI database for the Japanese quail (organism id: 93,934, version 2019-10-18) [53] to perform functional analysis at the gene level. The Gene Ontology enrichment was explored with the R package ViSEAGO [27] with the whole quail genome as background. Enrichment tests were performed on each list of DEG from females and males datasets using exact Fisher’s test. Enriched GO terms are provided in the Additional file 7. All enriched GO terms (*p* ≤ 0.01) were grouped into functional clusters using hierarchical clustering based on Wang’s semantic similarity between GO terms respecting GO graph topology and Ward’s criterion.

## Supplementary Information

**Additional file 1.** 

**Additional file 2.**

**Additional file 3.**

**Additional file 4.**

**Additional file 5.**

**Additional file 6.**

**Additional file 7.**

**Additional file 8.**

## Data Availability

The datasets supporting the conclusions of this article are available at the GEO repository GSE161976 https://www.ncbi.nlm.nih.gov/geo/query/acc.cgi?acc=GSE161976.

## Abbreviations

TM: Thermal Manipulation
C: Control
HC: Heat Challenge
RT: Room Temperature
TMHC: TM followed by a HC treatment
TMRT: TM followed by a RT treatment
CHC: C followed by a HC treatment
CRT: C followed by a RT treatment
DEG: Differentially expressed gene
RNA: Ribonucleic acid
DNA: Deoxyribonucleic acid
ET: Embryonic treatment
D35: Day 35
RT-qPCR: Reverse transcription followed by quantitative polymerase chain reaction
GO: Gene ontology
vs: Versus
