# Supplementary material for: Embryonic thermal manipulation impacts the postnatal transcriptome response of heat-challenged Japanese quails
Source: BMC Genomics. 2021 Jun 30;22:488. doi: 10.1186/s12864-021-07832-7 (PMC8243606; doi:10.1186/s12864-021-07832-7)

Additional file 4: MA plots of the differential analysis. Numbers indicated in red or blue represent the numbers of differentially expressed genes (DEG) obtained in each pairwise comparison; upregulated gene numbers are shown in red, downregulated genes numbers are shown in blue. Male results are shown in the left-hand side of the figure, female results in the righ-hand side. CRT: Control incubation followed by a room temperature treatment at D35; CHC: Control incubation followed by a heat challenge treatment at D35; TMRT: Thermal manipulation during incubation followed by a room temperature treatment at D35; TMHC: Thermal manipulation during incubation followed by a heat challenge treatment at D35.

Males	Females

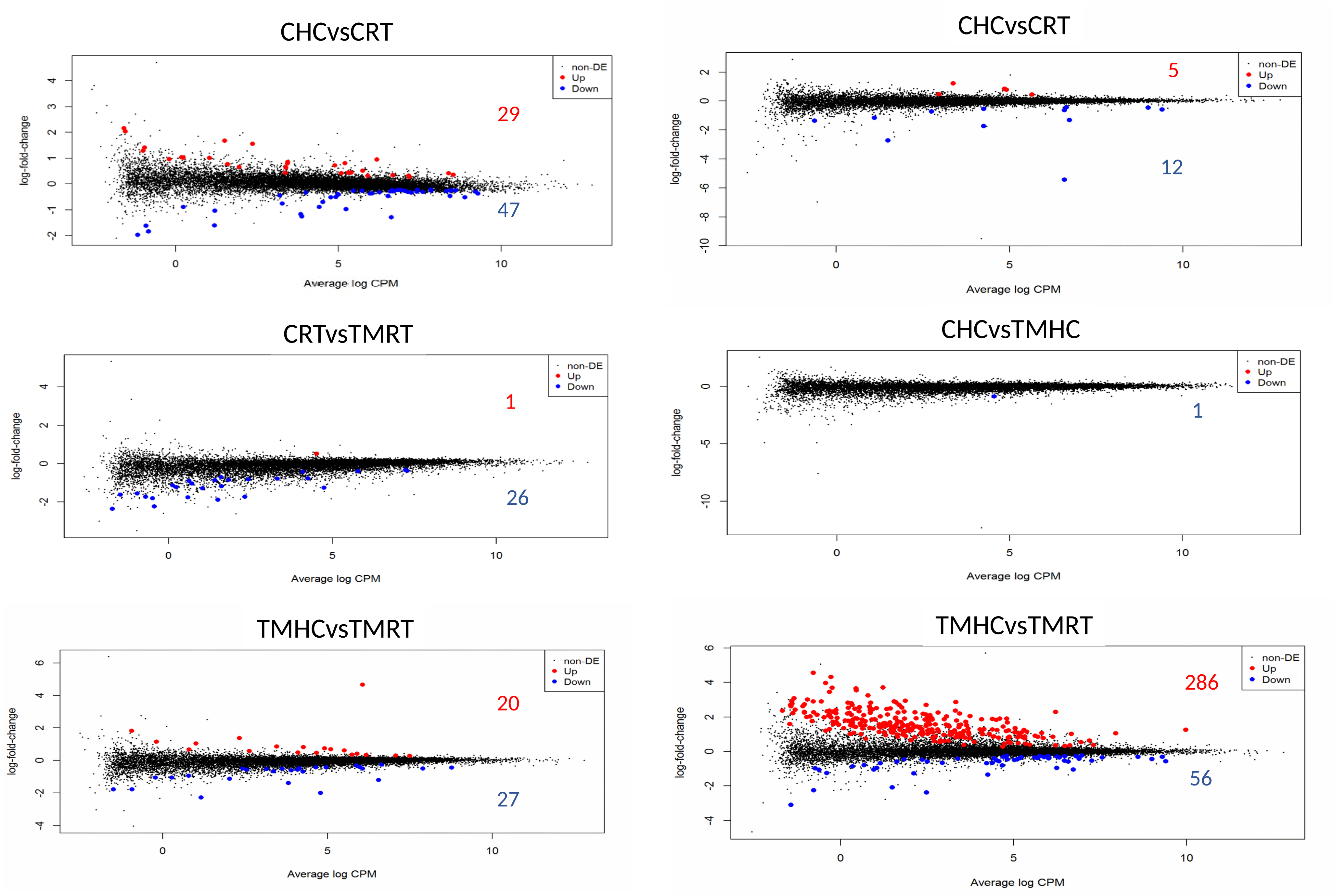

Supplement: Supplementary file 4 — Additional file 4. [file 12864_2021_7832_MOESM4_ESM.pdf]
