# Supplementary material for: Embryonic thermal manipulation impacts the postnatal transcriptome response of heat-challenged Japanese quails
Source: BMC Genomics. 2021 Jun 30;22:488. doi: 10.1186/s12864-021-07832-7 (PMC8243606; doi:10.1186/s12864-021-07832-7)

Additional file 5: MDS plots. (a) MDS plot for the female samples. (b) MDS plot for the male samples. CRT (green circles): Control incubation followed by a room temperature treatment at D35; CHC (squares with blue lines): Control incubation followed by a heat challenge treatment at D35; TMRT (blue squares filled): Thermal manipulation during incubation followed by a room temperature treatment at D35; TMHC (red triangles): Thermal manipulation during incubation followed by a heat challenge treatment at D35. The two TMHC females that display similarity to the TMRT females are circled in red in (a).

(a) females

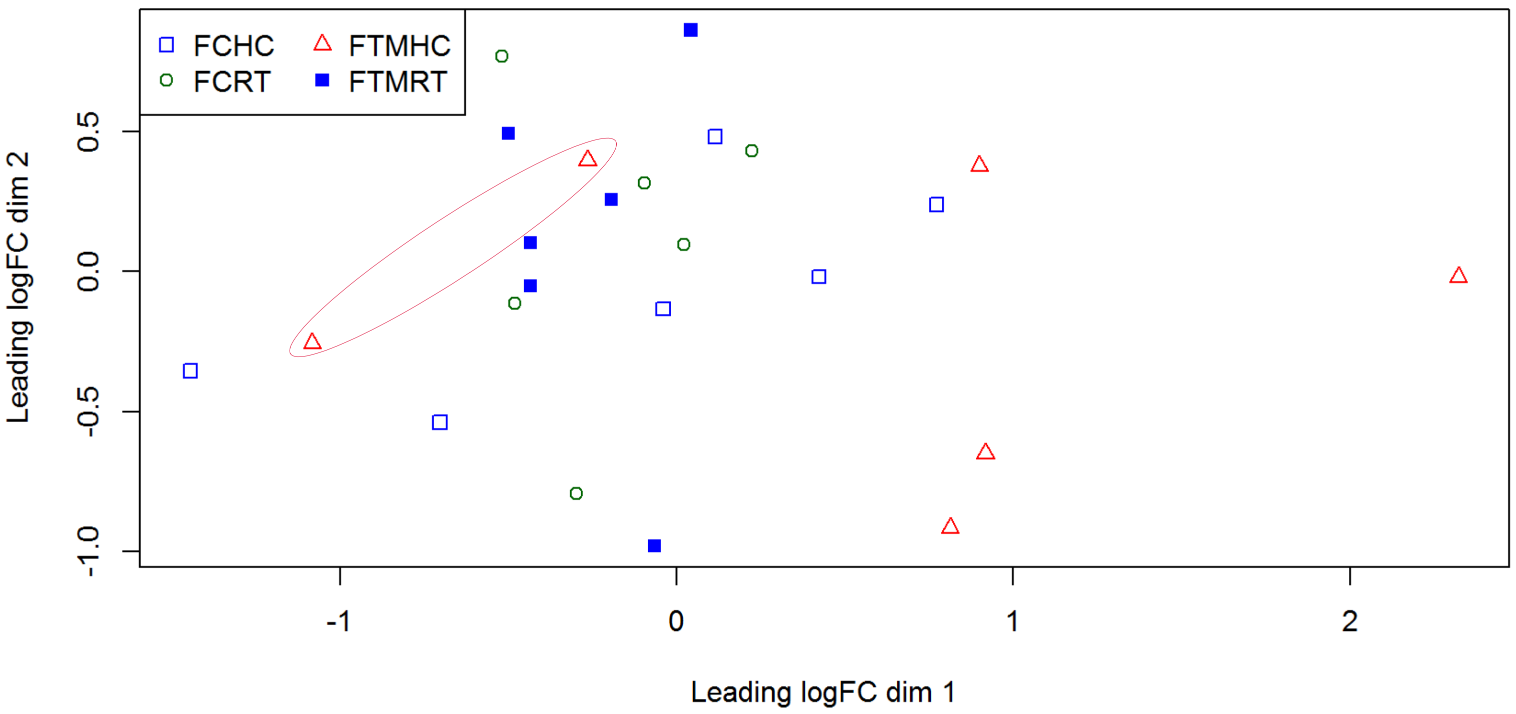

(b) males

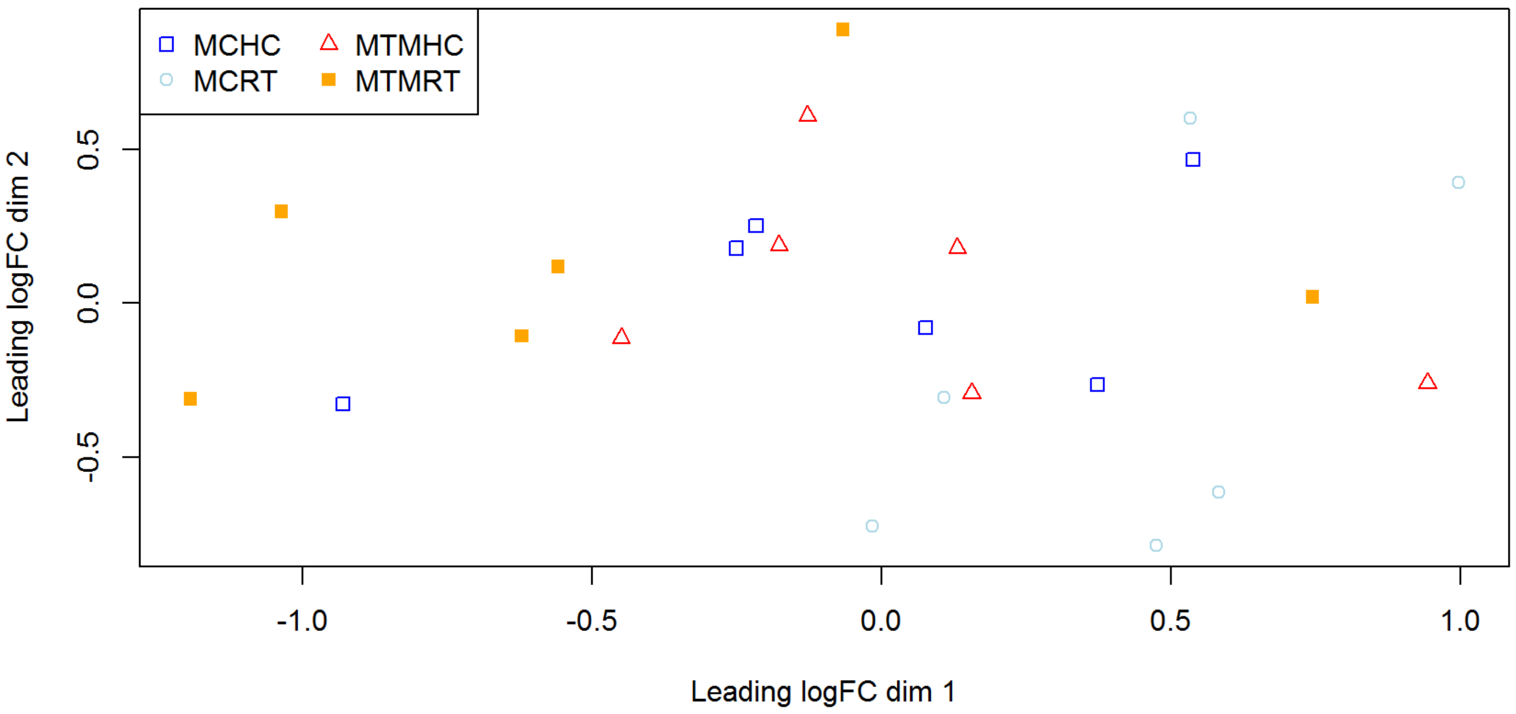

Supplement: Supplementary file 5 — Additional file 5. [file 12864_2021_7832_MOESM5_ESM.pdf]
