# Supplementary material for: Embryonic thermal manipulation impacts the postnatal transcriptome response of heat-challenged Japanese quails
Source: BMC Genomics. 2021 Jun 30;22:488. doi: 10.1186/s12864-021-07832-7 (PMC8243606; doi:10.1186/s12864-021-07832-7)

Additional file 6: Venn diagram representing the overlap of differentially expressed genes (DEG) between males and females for CHC vs CRT and TMHC vs TMRT comparisons. Gene names of DEG found in at least two comparisons are shown. CRT: Control incubation followed by a room temperature treatment at D35; CHC: Control incubation followed by a heat challenge treatment at D35; TMRT: Thermal manipulation during incubation followed by a room temperature treatment at D35; TMHC: Thermal manipulation during incubation followed by a heat challenge treatment at D35.

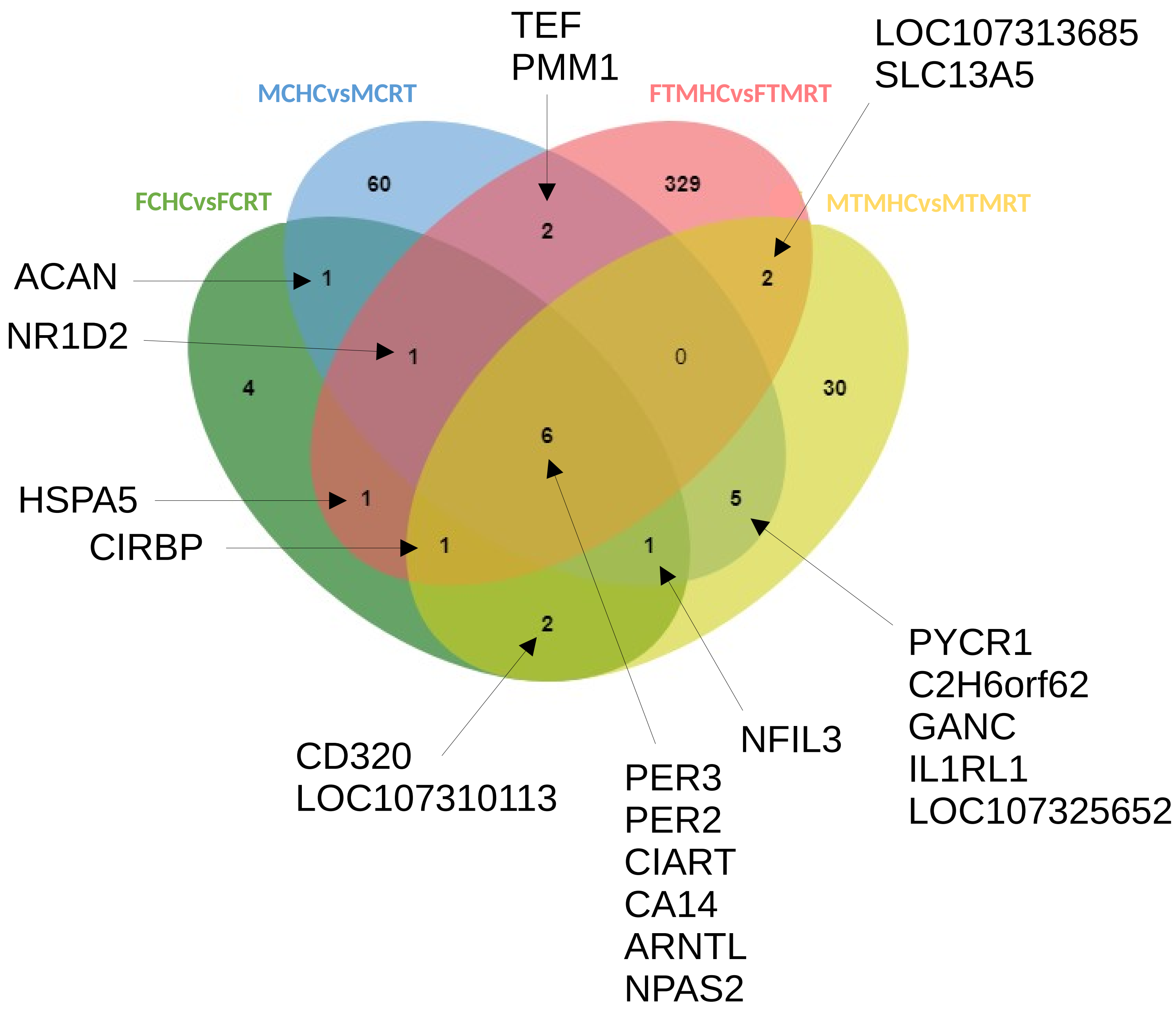

Supplement: Supplementary file 6 — Additional file 6. [file 12864_2021_7832_MOESM6_ESM.pdf]
